# Re-Visiting Antioxidant Therapy in Murine Advanced Atherosclerosis with Brussels Chicory, a Typical Vegetable in Mediterranean Diets

**DOI:** 10.3390/nu15040832

**Published:** 2023-02-06

**Authors:** Qing Li, Yushi Du, Panyin Xiang, Guanyu Chen, Xiaoxian Qian, Shuangshuang Li, Yihui Mao, Wenhua Ling, Dongliang Wang

**Affiliations:** 1 Department of Nutrition, School of Public Health, Sun Yat-sen University (Northern Campus), Guangzhou 510080, China; 2 Department of Cardiology, The Third Affiliated Hospital, Sun Yat-sen University, Guangzhou 510630, China; 3 Guangdong Provincial Key Laboratory for Food, Nutrition and Health, Guangzhou 510080, China

**Keywords:** Brussels chicory, advanced atherosclerosis, oxidative stress, NADPH oxidase, uncoupled endothelial nitric oxide synthase, glutathione

## Abstract

Brussels chicory, a typical vegetable in Mediterranean diets, has been recently reported to stabilize advanced atherosclerotic plaques in the brachiocephalic artery of apoE-deficient (*Apoe*^−/−^) mice. Herein, we investigated whether Brussels chicory can stabilize advanced plaques in the aorta via improving oxidative stress. Thirty week old *Apoe*^−/−^ mice were fed the AIN-93G diet or supplemented with 0.5% freeze-dried Brussels chicory for twenty weeks. Aortic plaque size and stability, aortic relaxation, monocyte adhesion to aortic endothelium, free radicals, and enzymatic and non-enzymatic factors involved in free radical production and elimination in aorta and serum were measured. Brussels chicory consumption did not alter aortic plaque size, however, it stabilized aortic plaques, promoted aortic relaxation, and also inhibited monocyte adhesion to aortic endothelium. Moreover, this administration reduced oxidized LDL (ox-LDL) and 4-hydroxynonenal (4-HNE) content in aortic plaques, associated with inhibited aortic NADPH oxidase (NOX) and uncoupled endothelial nitric oxide synthase (eNOS)-mediated free radical production. However, Brussels chicory consumption did not appreciably alter aortic and serum superoxide dismutase (SOD), catalase (CAT), and glutathione peroxidase (GPx) activities, aortic glutathione (GSH), as well as serum non-enzymatic antioxidants, such as bilirubin, uric acid, and GSH. Collectively, improved oxidative stress might contribute to the atheroprotective effect of Brussels chicory, supporting the prospect of the antioxidant therapy in advanced atherosclerosis progression.

## 1. Introduction

Cardiovascular diseases are the leading global cause of death, with atherosclerosis being a major contributor [1]. Adherence to a Mediterranean dietary pattern is known to inhibit atherosclerosis [2]. Our group recently observed that Brussels chicory (*Cichorium intybus* L. var. *foliosum*, *Belgian endive*), a typical Mediterranean vegetable, could stabilize advanced atherosclerotic plaques in the brachiocephalic artery of a apolipoprotein E-deficient (*Apoe*^−/−^) mouse model [3]. As atherosclerosis affects very specific sites of the vasculature [4], it remains unknown whether Brussels chicory stabilizes advanced atherosclerotic plaques in the aorta, the major site of atherosclerosis in vivo.

Persistent oxidative stress is vital for the etiology and progression of atherosclerosis [5]. Oxidative stress occurs when the production of free radicals, namely, reactive oxygen and nitrogen species, overwhelms their elimination [6]. The predominant sources of free radical production in arterial walls are NADPH oxidase (NOX), xanthine oxidase (XO), and uncoupled endothelial nitric oxide synthase (eNOS) in the context of atherosclerosis [5]. On the other hand, antioxidant enzymes and non-enzymatic antioxidants eliminating free radicals in arterial walls principally consist of superoxide dismutase (SOD), catalase (CAT), and glutathione peroxidase (GPx); and glutathione (GSH), respectively [5]. However, clinical trials with classic vitamin antioxidants failed to demonstrate any benefits in cardiovascular outcomes [7]. While a definite cause-and-effect relationship between oxidative stress and atherosclerosis in humans has not yet been established [8], the prospect of improving oxidative stress in the prevention and treatment of atherosclerosis remains enticing.

Chicory (*Cichorium intybus* L., Asteraceae) has a long-standing medicinal use dating back to the ancient Egyptian times [9,10]. Chicory is currently an important part of the human diet as a substitute for coffee and also as a major source of inulin, as well as use as a vegetable with its cultivated types, such as Brussels chicory, radicchio, and puntarelle [11]. Modern experimental studies have shown that chicory possesses a variety of pharmaceutical properties, ranging from antiulcerogenic, diuretic, analgesic, and wound-healing effects to an anti-metabolic disease potential (e.g., anti-diabetes, lowering blood pressure, and immunomodulatory effect) [9,11,12]. Moreover, our groups found that, in spite of no change in advanced atherosclerotic plaque size at the site of the brachiocephalic artery, Brussels chicory did elicit a “clinically favorable” stable-plaque phenotype with a reduction in necrotic size and an increase in collagen content and also the thickness of thin fibrous cap in *Apoe*^−/−^ mice [3]. Interestingly, the stable-plaque phenotype was associated with no changes in serum cholesterol and triglyceride levels. Instead, its benefit may result, at least partly, from reshaped gut microbiota profiles, inhibited gut microbiota lipopolysaccharide production, and reduced intestinal permeability [3].

Brussels chicory is rich in polyphenols, especially phenolic acid that is up to around 200 mg per 100 g fresh weight, appropriately 10–100-fold higher than those in other common vegetables such as onion and broccoli [13,14,15]. Dietary supplementation of ferulic acid, chicoric acid, or protocatechuic acid that are abundant in Brussels chicory has been reported to appreciably improve oxidative stress at tissue and organ levels in the context of varied types of metabolic diseases, likely through direct interactions with receptors or enzymes linked to oxidative stress production and elimination rather than direct scavenging free radicals [16,17,18]. Supporting this probability, dietary supplementation of Brussels chicory did not affect serum total antioxidant capabilities and oxidized LDL (ox-LDL) levels in *Apoe*^−/−^ mice in the context of either early or advanced atherosclerosis [3,19]. Nevertheless, there are no data showing the effect of Brussels chicory on oxidative stress in arterial walls with atherosclerotic plaques by targeting receptors or enzymes involved in oxidative stress production and elimination. 

## 2. Materials and Methods

### 2.1. Mouse Study

The procedures of mouse studies and mouse tissue samples were adopted from our previous studies [3]. Thirty week old male *Apoe*^−/−^ mice on the C57BL/6J background (Vital River Laboratory Animal Technology Corporation, Beijing, China) with advanced atherosclerotic plaques [20] were fed an AIN-93G diet supplemented without or with 0.5% (wt:wt) of freeze-dried Brussels chicory (Hebei Vilof Agritech Co., Ltd., Beijing, China). A total of 15 mice per group were used. The composition of experimental diets was detailed in Table S1 in the Supplementary Information. Mice aged 50 weeks were then sacrificed, followed by harvesting different parts of aortas (aortic root, ascending aorta, and aortic arch, as well as thoracic and abdominal aorta) and blood samples for further analyses. 

### 2.2. Advanced Plaque Size and Stability

Advanced plaque size and stability were assessed by either hematoxylin and eosin or Sirius red staining as we previously described [3]. Serial frozen sections of heart (8 μm) were stained with either hematoxylin and eosin for the measurement of plaque size, necrotic core size, and thickness of thin fibrous cap, or Sirius red for the quantification of collagen content. All images of the stained samples were captured by an operator blinded to the experimental groups by using a Leica DM2500 LED optical microscope. Quantification of plaque area occupied by a particular stain was expressed as a percentage of plaque size with Image Pro plus Software 7.0 (Media Cybernetics, Rockville, MD, USA). 

### 2.3. Cellular and Molecular Components of Advanced Plaque

Cellular and molecular compositions of advanced plaques were analyzed using immunohistochemistry of frozen sections of heart as we previously documented [21]. Primary antibodies used were rat polyclonal anti-CD68 (14-0681-82, Invitrogen, Waltham, MA, USA), rabbit polyclonal anti-actin α 2 (ACTA2; PA5-85070, Invitrogen), and rabbit polyclonal anti-cleaved CASP3 (9664, Cell Signaling Technology, Danvers, MA, USA). The omission of primary antibodies was considered a negative control. Neutral lipid in advanced plaques was analyzed by oil red O staining [21]. All images of the stained samples were captured by an operator blinded to the experimental groups. The area occupied by a particular stain was shown as a percentage of plaque size.

### 2.4. Aortic Relaxation

Aortic relaxation was measured with acetylcholine (Ach, A6625, Sigma-Aldrich, St. Louis, MO, USA) or sodium nitroprusside (SNP, 71778, Sigma-Aldrich) as we previously described [22]. A 3 mm long ring of the top thoracic aorta was attached to a force transducer and suspended in an organ bath filled with Krebs–Ringer solution (pH 7.4). The ring was progressively subjected to a predetermined optimal tension of 30 mN, at which point the vessel ring was allowed to equilibrate for at least half an hour. Next, the ring was precontracted with 1 μmol/L phenylephrine (P1240000, Sigma-Aldrich) for 60–90 min, followed by exposure to various concentrations of Ach to measure endothelium-dependent relaxation. Endothelial-independent relaxation as a result of exposure to various concentrations of SNP was also examined. To evaluate endothelial-dependent relaxation, rings were pre-treated with 300 μmol/L Nω-nitro-L-arginine methyl ester (L-NAME, N5751, Sigma-Aldrich) for 30 min.

### 2.5. Detection of Monocyte Adherence to Aortic Endothelium

The ex vivo adhesion of monocyte to the ascending aortic endothelium was evaluated as we previously described [23]. In brief, the ascending aortas were opened longitudinally and incubated with 1 × 10^6^ calcein-acetoxymethyl-ester-labeled HL-60 monocytes for 30 min. After incubation, unbound HL-60 cells were removed by ice-cold PBS. Images were captured with Leica TCS SP5 laser scanning confocal microscope by two observers blind to the experimental groups, and the number of adherent monocytes were then counted in 5 consistent fields. 

### 2.6. Oxidative Stress Burden in Advanced Plaque

Oxidative stress in advanced plaques was evaluated by fluorescent dihydroethidium (DHE) staining as previously described [24]. Frozen sections of aortic root were incubated with 2 μmol/L DHE (D11347, Thermo Fisher Scientific, Waltham, MA, USA) at 37 °C for 30 min. Images were captured by confocal microscope and the mean intensity of fluorescent signal per plaque area was then quantified.

### 2.7. Oxidative Products ox-LDL and 4-Hydroxynonenal (4-HNE) in Advanced Plaque

Ox-LDL and 4-HNE in advanced plaques of frozen tissue sections were analyzed using immunohistochemistry assays. Primary antibodies used were rabbit polyclonal anti-ox-LDL (bs-1698R, Bioss, Woburn, MA, USA) and mouse monoclonal anti-4-HNE (MA5-27570, Invitrogen). The omission of primary antibodies was used as negative controls. Images of the stained samples were captured by an operator blinded to the experimental groups. The area occupied by a particular stain was shown as a percentage of plaque size. 

### 2.8. Measurement of Superoxide

Superoxide levels in thoracic and abdominal aorta rings were assessed by lucigenin-enhanced chemiluminescence as previously documented [25]. Samples were added to polypropylene cuvettes with PBS and lucigenin (5 μmol/L). After 10 min of dark adaptation, basal chemiluminescence was measured for 5 min using a luminometer (FB15, Zylux, Maryville, TN, USA). For measurement of NOX activity, luminescence was assessed using NADH (0.2 and 1 mmol/L, ST358, Beyotime, Shanghai, China) or NADPH (0.2 and 1 mmol/L, ST360, Beyotime) as substrates. The source of superoxide was determined by treating samples with apocynin (targeting NOX, 100 µmol/L, 178385, Sigma-Aldrich), L-NAME (targeting eNOS, 100 µmol/L) or oxypurinol (targeting XO, 100 µmol/L, O6881, Sigma-Aldrich) for 30 min prior to chemiluminescence measurement. Relative light unit (RLU) values were normalized to the surface area of each vessel. 

### 2.9. Aortic Oxidant ENZYME Activity

The eNOS activity was determined using eNOS assay kit (A014, Nanjing Jiancheng Bioengineering Institute, Nanjing, China), following the manufacturer’s instructions. The XO activity in aortas were measured by pterin-based method [26]. In brief, the supernatants of aorta tissue homogenates were incubated with 50 μM pterin (P1132, Sigma-Aldrich) for 1 h at 37 °C. Activity was then measured as units/mg protein in aortas with buttermilk XO (682151, Merck Millipore, Burlington, MA, USA) as standard.

### 2.10. Aortic and Serum Antioxidant Enzyme Activity

The SOD activity was determined using WST-1 method with commercial kit, obtained from Nanjing Jiancheng Bioengineering Institute (A001-3), according to the manufacturer’s protocol. The GPx activity was assessed with spectrophotometry as previously described [27]. Briefly, the prepared supernatants of aorta homogenates or serum samples were added to the GSH/NADPH/glutathione reductase system. The reaction was initiated by the addition of 4 mM H_2_O_2_. The GPx activity was evaluated using an NADPH decay and expressed as units/mg protein in aortas or units/mL in serum. The CAT activity was assayed using the protocol of Aebi [28] by monitoring the H_2_O_2_ decomposition. In brief, the protein samples were added to phosphate buffer containing H_2_O_2_ and the consumption of H_2_O_2_ was then spectrophotometrically measured at 240 nm.

### 2.11. qRT-PCR

Total RNA was isolated from aortas using TRI reagent (AM9738, Invitrogen). RNA concentration measurement was based on the absorbance at 260 nm. For mRNA quantification, RNA was reverse-transcribed to cDNA using QuantiTect Reverse Transcription Kit (205311, Qiagen, Hilden, Germany), followed by quantitative real-time PCR with SYBR Green PCR Master Mix (4309155, Thermo Fisher) performed on ABI 7300 Real-Time qPCR System. β-actin was used as an internal control. The primer sequences of indicated genes were listed in Table S2 in the Supplementary Information.

### 2.12. Aortic and Serum Non-Enzymatic Antioxidants

The GSH and oxidized GSH (GSSG) level in aorta and serum were measured by a commercial kit (S0053, Beyotime, Shanghai, China). The albumin and uric acid level in serum was assayed using mice serum albumin ELISA kit (D721206, Sangon Biotech, Shanghai, China) and uric acid assay kit (ab65344, Abcam, Cambridge, UK), respectively. The vitamin C, vitamin E, and total bilirubin level in serum were assayed using corresponding colorimetric assay kit (E-BC-K034, E-BC-K033, E-BC-K760, Elabscience, Houston, TX, USA). 

### 2.13. Statistical Analyses

Data were expressed as the means ± SD. Normality of the data were tested by the Kolmogorov–Smirnov test. The data were analyzed by a two-tailed unpaired Student’s t-test with or without the Welch correction performed on GraphPad Prism Version 9.3.1. Statistical significance was accepted for *p* < 0.05.

## 3. Results

### 3.1. Brussels Chicory Stabilizes Atherosclerotic Plaques at the Site of Aortas

**Figure 1:**
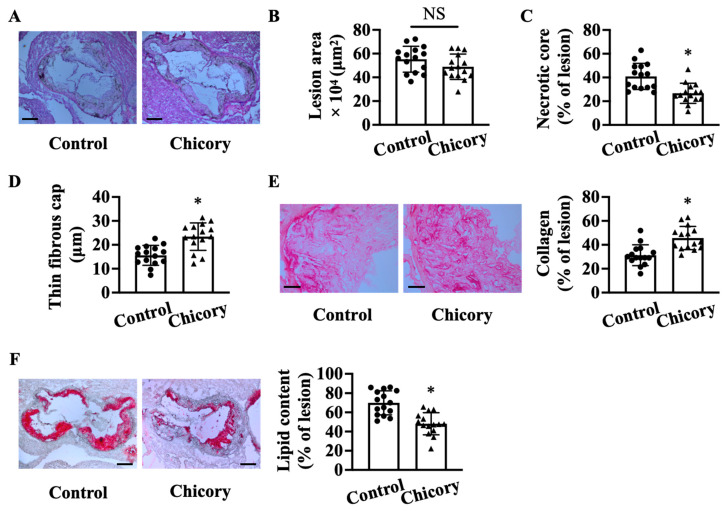
Brussels chicory consumption improves plaque stability without affecting plaque size in the aortic roots of *Apoe*^−/−^ mice. (**A**) Representative images of hematoxylin and eosin staining. Scale bars: 250 μm. (**B**–**D**): plaque size (**B**), necrotic core size (**C**), thin-fibrous cap thickness (**D**) in plaques of the aortic root. (**E**) Representative images of Sirius red staining (left panel) and quantification (right panel) of collagen accumulation in plaques. Scale bars: 50 μm. (**F**) Representative images of oil red O staining (left panel) and quantification (right panel) of the neutral lipid content in plaques. Scale bars: 250 μm. Data are presented as means ± SD. *n* = 15. * Significantly different from the control group (*p* < 0.05). Apoe, apolipoprotein E; Chicory, Brussels chicory; NS, nonsignificant (*p* ≥ 0.05).

Morphometric analyses show that Brussels chicory consumption for 20 weeks does not significantly affect aortic root plaque size as compared with the control treatment (Figure 1A,B). Instead, this administration improves several features of plaque stability, including decreased necrotic core size by 34.8% (Figure 1C), increased thickness of thin fibrous cap by 25.5% (Figure 1D), and collagen content by 31.6% (Figure 1E), and reduced neutral lipid content by 31.3% (Figure 1F).

**Figure 2:**
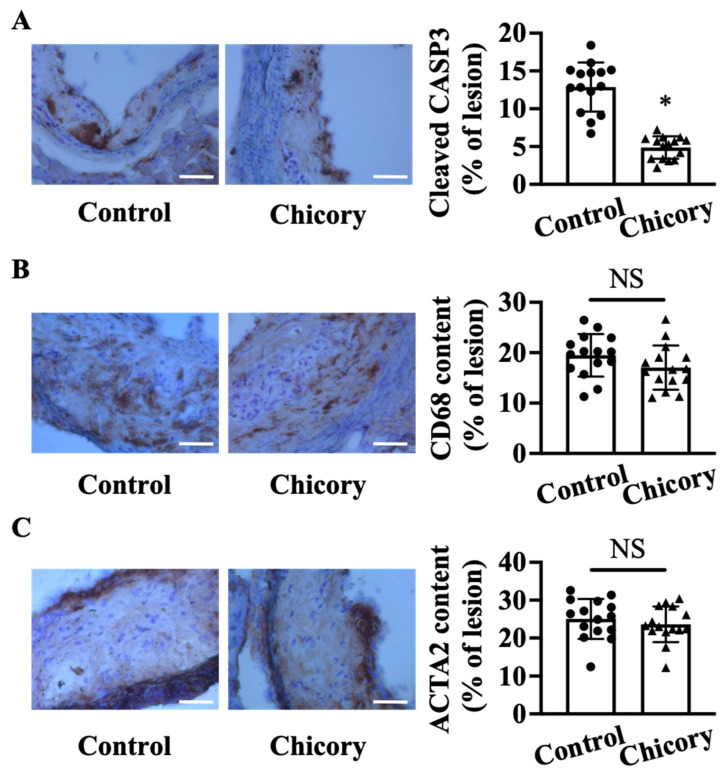
Brussels chicory consumption reduces plaque apoptosis without affecting the content of macrophages and smooth muscle cells in aortic root plaques in *Apoe*^−/−^ mice. (**A**–**C**) Representative immunohistochemistry images of cleaved CASP3 ((**A**), left panel), CD68 ((**B**), left panel), and ACTA2 ((**C**), left panel) in aortic root sections and quantification of the percentage of cleaved CASP3 ((**A**), right panel), CD68 ((**B**), right panel), and ACTA2 positive cells ((**C**), right panel) in aortic root plaques. Scale bar, 100 μm. Data are presented as means ± SD. *n* = 15. * Significantly different from the control group (*p* < 0.05). Scale bars: 100 μm. ACTA2, actinα2; Apoe, apolipoprotein E; Chicory, Brussels chicory; NS, nonsignificant (*p* ≥ 0.05).

At the cellular component level, Brussels chicory consumption reduces cellular apoptosis by 62.3% in aortic root plaques (*p* < 0.05; Figure 2A). The number of CD68^+^ (a common marker for macrophages) and ACTA2^+^ (a common marker for vascular smooth muscle cells) cells (Figure 2B,C) in aortic root plaques are comparable between the tested groups. Consistently, the ratio of macrophages to vascular smooth muscle cells in the Brussels chicory group (0.760 ± 0.273) is not significantly different from the control group (0.799 ± 0.171).

### 3.2. Brussels Chicory Improves Aortic Endothelium Functions

**Figure 3:**
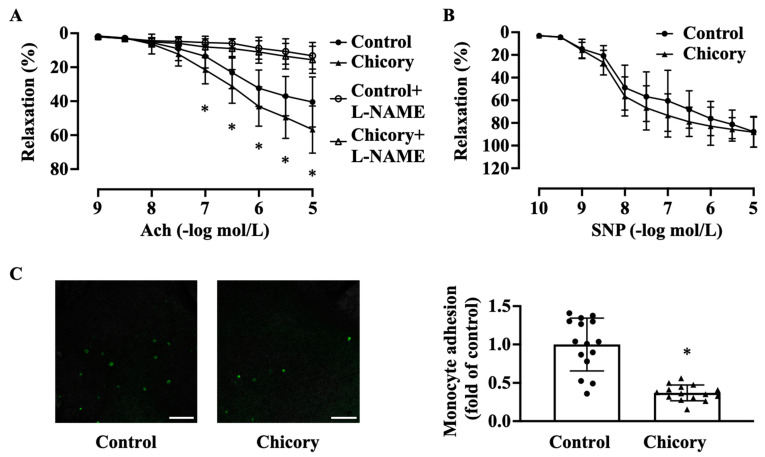
Brussels chicory consumption improves aortic endothelial-dependent relaxation and inhibits monocyte adhesion to aortic endothelium in *Apoe*^−/−^ mice. (**A**) Thoracic aortic endothelial dependent relaxation in response to acetylcholine (Ach) in the presence or absence of Nω-nitro-L-arginine methyl ester (L-NAME). (**B**) Thoracic aortic endothelial-independent relaxation in response to sodium nitroprusside (SNP). (**C**) Representative images (left panel) and quantification (right panel) of the adhesion of green fluorescent calcein-labeled HL-60 monocytes to the isolated endothelium of ascending aortas. Scale bars: 250 μm. Data are presented as means ± SD. *n* = 15. * Significantly different from the control group (*p* < 0.05). Apoe, apolipoprotein E; Chicory, Brussels chicory.

Brussels chicory consumption promotes endothelium-dependent relaxation to Ach (Figure 3A). This effect seems to be specific as this administration does not affect endothelium-independent relaxation to SNP (Figure 3B). Moreover, Brussels chicory consumption attenuates the adherence of green fluorescent calcein-labeled monocytes to ascending aortic endothelium (Figure 3C).

### 3.3. Brussels Chicory Alleviates Oxidative Stress in Atherosclerotic Plaques

**Figure 4:**
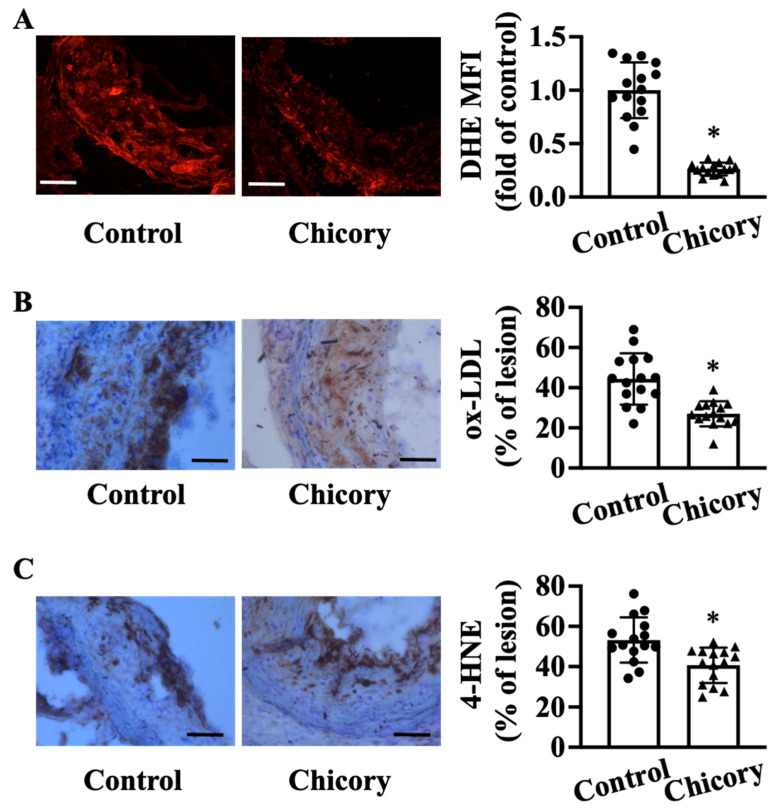
Brussels chicory consumption improves oxidative stress in atherosclerotic plaques in *Apoe*^−/−^ mice. (**A**) Representative images of dihydroethidium (DHE) staining (left panel) and quantification of the fluorescence signals (right panel) in plaques of the aortic root. Scale bars: 100 μm. (**B**,**C**) Representative immunohistochemistry images of oxidative products ox-LDL ((**B**), left panel) and 4-HNE ((**C**), left panel) in aortic root sections and quantification of the ox-LDL ((**B**), right panel) and 4-HNE positive area ((**C**), right panel). Scale bar, 100 μm. Data are presented as means ± SD. *n* = 15. * Significantly different from the control group (*p* < 0.05). Apoe, apolipoprotein E; Chicory, Brussels chicory; MFI, mean fluorescence intensity.

Compared with the control treatment, Brussels chicory consumption elicits a significant reduction in oxidative stress in aortic root plaques, as indicated by a reduction in O2^·−^ levels assessed by DHE staining (Figure 4A). Concordantly, this administration decreases the content of oxidative products ox-LDL by 39.15% (Figure 4B) and 4-HNE by 23.6% (Figure 4C) in aortic root plaques (both *p* < 0.05).

### 3.4. Brussels Chicory Inhibits NADPH Oxidase- and Uncoupled eNOS-Mediated Oxidative Stress Production in Atherosclerotic Plaques

**Figure 5:**
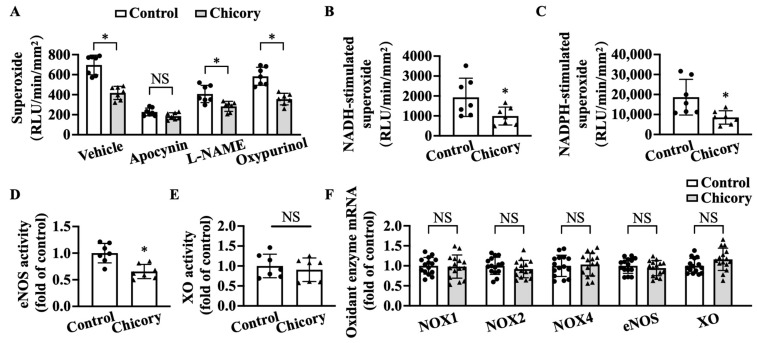
Brussels chicory alleviates NADPH oxidase (NOX)- and uncoupled endothelial nitric oxide synthase (eNOS)-mediated free radical production in the aortas of *Apoe*^−/−^ mice. (**A**) Thoracic and abdominal aortic superoxide production in the presence or absence of either apocynin, Nω-nitro-L-arginine methyl ester (L-NAME) or oxypurinol (n = 7). (**B**,**C**) NADH-stimulated (**B**) and NADPH-stimulated (**C**) superoxide levels in thoracic and abdominal aortas (n = 7). (**D**,**E**) Relative eNOS (**D**) and XO (**E**) activities in the thoracic and abdominal aortas (n = 7). (**F**) Relative NADPH oxidases, eNOS, and XO expression in the aortic arches at the mRNA level measured by qRT-PCR (*n* = 15). Data are presented as means ± SD. * Significantly different from the control group (*p* < 0.05). Scale bars: 100 μm. Apoe, apolipoprotein E; Chicory, Brussels chicory.

Brussels chicory consumption significantly reduces basal levels of O2^·−^ in thoracic and abdominal aortas, whereas apocynin (a specific inhibitor for NOX) but not L-NAME (a specific inhibitor for eNOS) and oxypurinol (a specific inhibitor for XO) abolish the inhibitory effect of Brussels chicory on thoracic and abdominal aortic O2^·−^ production (Figure 5A). The addition of NADH or NADPH increases O2^·−^ levels in tested groups, where the Brussels chicory group shows decreased O2^·−^ generation in response to NADH or NADPH stimulation, compared with the control group (Figure 5B,C). Of note, Brussels chicory consumption also decreases uncoupled eNOS activity without affecting XO activity (Figure 5D,E). qRT-PCR analyses further confirm that Brussels chicory consumption does not affect NOX, eNOS, and XO expression at the mRNA levels (Figure 5F).

### 3.5. Brussels Chicory Does Not Affect Enzymatic and Non-Enzymatic Antioxidant System in Atherosclerotic Plaques

**Figure 6:**
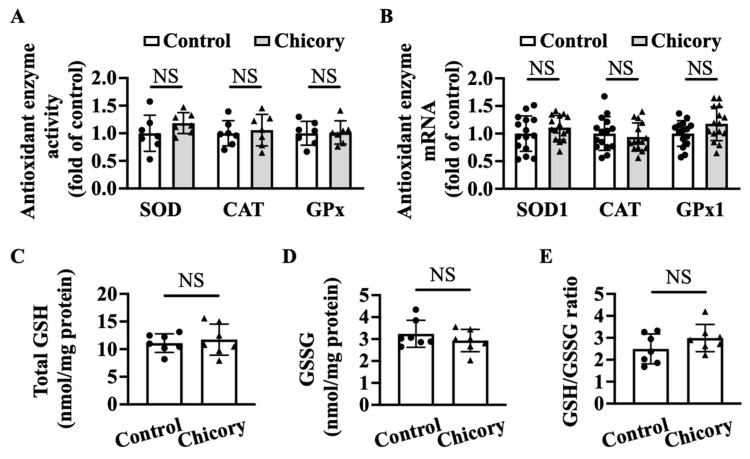
Brussels chicory does not alter enzymatic and non-enzymatic molecules with antioxidant function in the aortas of *Apoe*^−/−^ mice. (**A**) Antioxidant activities of superoxide dismutase (SOD), catalase (CAT,) and glutathione peroxidase (GPx) in thoracic and abdominal aortas (*n* = 7). (**B**) Relative SOD1, CAT, and GPx1 expression in the aortic arches at the mRNA level assessed by qRT-PCR (*n* = 15). (**C**–**E**) Total glutathione (GSH) (**C**) and oxidized GSH (GSSG) (**D**) content and the ratio of reduced GSH to GSSG (**E**) in thoracic and abdominal aortas (*n* = 7). Data are presented as means ± SD. Apoe, apolipoprotein E; Chicory, Brussels chicory; NS, nonsignificant (*p* ≥ 0.05).

Brussels chicory consumption does not appreciably affect thoracic and abdominal aortic SOD, CAT, or GPx activities as compared with the control treatment (Figure 6A). qRT-PCR analyses further confirm that Brussels chicory consumption does not affect aortic *Sod1*, *Cat*, and *Gpx1* mRNA levels (Figure 6B). Moreover, Brussels chicory consumption does not change the content of total GSH, GSSG content or the ratio of GSH/GSSG in thoracic and abdominal aortas (Figure 6C–E).

### 3.6. Brussels Chicory DOES Not Alter Enzymatic and Non-Enzymatic Antioxidant System in Blood Circulation

**Figure 7:**
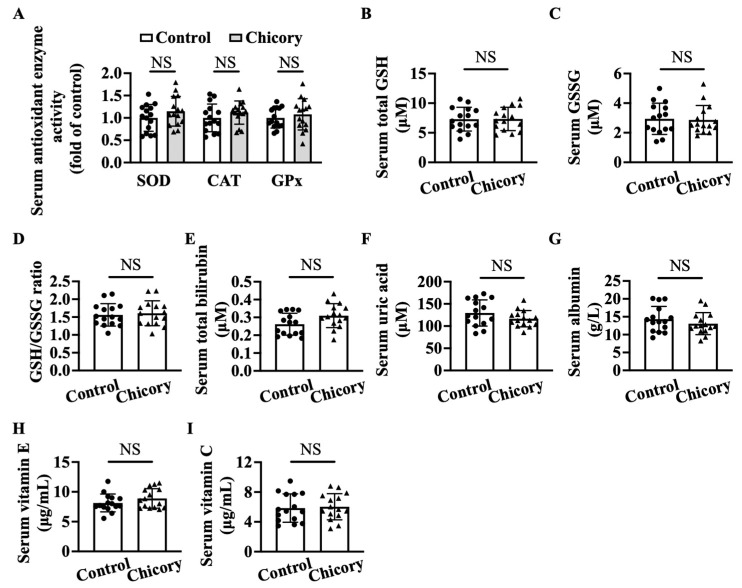
Brussels chicory does not alter enzymatic and non-enzymatic molecules with antioxidant function in the serum of *Apoe*^−/−^ mice. (**A**) Antioxidant activities of serum superoxide dismutase (SOD), catalase (CAT), and glutathione peroxidase (GPx). (**B**–**I**) Total glutathione (GSH) (**B**) and oxidized GSH (GSSG) (**C**) content, the ratio of reduced GSH to GSSG (**D**), total bilirubin (**E**), uric acid (**F**), albumin (**G**), vitamin E (**H**), and vitamin C (**I**) in serum. Data are presented as means ± SD. *n* = 15. Scale bars: 100 μm. Apoe, apolipoprotein E; Chicory, Brussels chicory. NS, nonsignificant (*p* ≥ 0.05).

Brussels chicory consumption does not appreciably affect serum SOD, CAT, or GPx activities as compared with the control treatment (Figure 7A). This administration also does not change the content of total GSH, GSSG, or the ratio of GSH/GSSG, total bilirubin, uric acid, albumin, vitamin E, and vitamin C (Figure 7B–I), the major non-enzymatic antioxidants in blood circulation.

## 4. Discussion

Oxidative stress is generally thought to be an important contributing factor in the development and progression of atherosclerosis, raising a rational therapeutic strategy that an antioxidant therapy could protect against atherosclerotic cardiovascular diseases [5]. Unexpectedly, most, if not all, clinical trials carried out so far have consistently shown that antioxidant vitamin regimens (vitamin E or vitamin A precursor β-carotene) or selenium do not prevent cardiovascular disease events, leading to a prevailing concept that the (dietary) antioxidant therapy is unable to counteract cardiovascular diseases [7,29,30,31,32]. Of note, a number of human observational studies have shown an inverse relationship between the whole food, plant-based dietary pattern rich in antioxidant polyphenols and the incidence of cardiovascular disorders [8,33,34]. More importantly, long-term dietary intervention trials recently demonstrated that adherence to a Mediterranean diet rich in polyphenols efficiently prevents cardiovascular events by ~30%, a comparable efficiency with statins [2,35]. Therefore, a whole food affluent in polyphenols rather than individual antioxidant vitamins might be a promising strategy to protect against cardiovascular diseases. 

Recently, we observed for the first time that dietary supplementation of Brussels chicory rich in phenolic acids for 20 weeks could stabilize advanced plaques in the brachiocephalic artery of *Apoe*^−/−^ mouse model [3]. Herein, we extend these findings to demonstrate that Brussels chicory consumption can also exert a stable-plaque effect in the aortic root. Concomitantly, Brussels chicory consumption improves aortic functions including an increase in endothelium-dependent relaxation and a reduction in the adherence of monocytes to endothelium. These benefits are in parallel with an improvement in oxidative stress in aortic plaques rather than systemic blood circulation. Mechanistic studies further uncovered that inhibited free radical production, through a reduction in NOX activities and reversing uncoupled eNOS, might contribute to the improved oxidative stress in aortic plaques. Our findings are, thus, reminiscent of the atheroprotective effect of a whole food, plant-based Mediterranean diet rich in polyphenols [8,33,34]. 

In addition to Brussels chicory, improving oxidative stress through dietary supplementation with individual polyphenols or polyphenols-rich extracts (e.g., anthocyanins [36,37], epigallocatechin gallate [38], protocatechuic acid [39], pomegranate peel extract [40]) or genetic modulation with increased GSH in macrophages [41] were associated with increased stability of advanced plaques in *Apoe*^−/−^ mice. More importantly, a small clinical trial study shows that drinking pomegranate juice rich in polyphenols can not only halt the progression of established atherosclerosis but actually reverse it [42]. Therefore, future research using either Brussels chicory, other dietary interventions rich in polyphenols or individual polyphenols should be considered to evaluate these effects on the stability of advanced atherosclerosis, the major factor determining the occurrence of cardiovascular disease events. 

The findings that Brussels chicory alleviates oxidative stress and concomitantly reduces the content of oxidant products (ox-LDL and 4-HNE) in aortic plaques prompted us to dissect the underlying mechanisms. Brussels chicory does not affect the expression and activities of aortic antioxidant enzymes SOD, CAT, or GPx, or the content of reduced GSH, an important endogenous non-enzymatic antioxidant. These observations strongly suggest that the oxidative stress-modulation effect of Brussels chicory inside aortic plaques is not due to enhanced free radical elimination. It should be pointed out that other antioxidant factors in aortic plaques, such as Nrf2 [43] and thioredoxin [44,45], might be involved, which we did not measure in the current study. On the other hand, Brussels chicory appreciably inhibits NOX to produce free radicals. Blocking NOX activity abolishes the oxidative-stress-modulation potency of Brussels chicory, suggesting that NOX is a major target for Brussels chicory. eNOS oxidizes L-arginine to L-citrulline and nitric oxide under normal physiological conditions, which vasodilates blood vessels, inhibits LDL oxidation, and also attenuates monocyte adherence to endothelium [46]. However, under pathological states including advanced atherosclerosis, eNOS is uncoupled and generates free radicals rather than nitric oxide [5]. Consistently, we observe that aortic root eNOS is uncoupled to produce free radicals in the control *Apoe*^−/−^ mice with advanced atherosclerosis, which is partly reversed by Brussels chicory administration (Figure 5). These findings are reminiscent of our previous findings that Brussels chicory consumption for 1 week reverses uncoupled eNOS and, in turn, promotes endothelium-dependent relaxation [22]. Consistently, Brussels chicory consumption for 20 weeks also promotes endothelium-dependent relaxation. Together, we favor the notion that Brussels chicory alleviates oxidative stress in aortic plaques, at least partly, by targeting NOX and uncoupled eNOS.

Increased free radical production and reduced antioxidant GSH bioavailability were demonstrated in experimental and human atherosclerosis [41,47]. Pharmacological and genetic studies further showed that increasing GSH protects against atherosclerosis development and vice versa [41,48,49]. These findings strongly suggest that increasing GSH is an effective approach to prevent cardiovascular disease events. Varì et.al reported that protocatechuic acid, one component of Brussels chicory, increased cellular GSH content in J774A.1 murine macrophages [50]. We also found that Brussels chicory consumption for 1 week increased GSH content in peritoneal macrophages derived from *Apoe*^−/−^ mice with advanced atherosclerosis [19]. Our current finding that Brussels chicory consumption for 20 weeks did not affect aortic reduced GSH content was, thus, unexpected. These inconsistencies could be partly explained by different intervention periods (20 weeks in the present study compared with 1 week previously) and different tissues/cells (aortas in the present study compared with peritoneal macrophages previously). As aortas with advanced atherosclerosis consist of different cell types (i.e., endothelial cells, smooth muscle cells, dendritic cells, Th1 cells, and other infiltrating immune cells), we cannot exclude the possibility that Brussels chicory did increase GSH content in macrophages within advanced plaques. This possibility is worthy to be tested because Brussels chicory and/or its component protocatechuic acid have been found to reduce the atherogenic capacity of macrophages within advanced plaques, including enhanced cholesterol efflux to extracellular ApoA-I and high-density lipoprotein and reduced inflammatory burden [19,39], both of which could be impaired by oxidative stress [51,52]. 

## 5. Conclusions

In conclusion, we showed that Brussels chicory can increase plaque stability in the aorta, at least in part through improving oxidative stress within plaques by inhibition of NOX and uncoupled eNOS-mediated free radical production in male *Apoe*^−/−^ mice. Together with other works [36,37,38,39,40,41], our findings allow us to call for a re-visiting to the potential of antioxidant therapy in preventing cardiovascular disease events.

## Data Availability

Data are contained within the article or Supplementary Material.

## Supplementary Materials

The following supporting information can be downloaded at: https://www.mdpi.com/article/10.3390/nu15040832/s1, Table S1: Composition of the experimental diets and freeze-dried Brussels chicory; Table S2: Primers for qRT-PCR.
