# Supplementary material for: Re-Visiting Antioxidant Therapy in Murine Advanced Atherosclerosis with Brussels Chicory, a Typical Vegetable in Mediterranean Diets
Source: Nutrients. 2023 Feb 6;15(4):832. doi: 10.3390/nu15040832 (PMC9966914; doi:10.3390/nu15040832)
Supplement: Supplementary file 1 [file nutrients-15-00832-s001.zip › nutrients-2180629-supplementary.pdf]

Supplementary Information

Table S1. Composition of the experimental diets and freeze-dried Brussels chicory.

	AIN-93G diet	Chicory-supplemented diet
Ingredient (g/kg)		
Freeze-dried Brussels chicory	0	5
protein		0.981
fat		0.155
carbohydrate		2.280
crude fiber		0.923
moisture		0.087
Phenolic acids		
protocatechuic acid		0.029
gallic acid		0.028
caffeic acid		0.003
5-caffeoylquinic acid		0.128
caftaric acid		0.020
chicoric acid		0.049
Sesquiterpene lactones		
lactucin		0.001
11β,13-dihydrolactucin		0.005
lactucopicrin		0.007
11β,13-dihydrolactucopicrin		0.0005
Casein	200	199.019
Corn starch	397.486	395.206
Maltodextrin	132	132
Sucrose	100	100
Soybean oil	70	69.845
Cellulose	50	49.077
AIN-93G mineral mix	35	35
AIN-93G vitamin mix	10	10
L-cystine	3	3
Choline bitartrate	2.5	2.5
Tert-butylhydroquinone	0.014	0.014

Table S2. Primers for qRT-PCR.

Genes	Primer sequences
Nox1 -Forward	GGTTGGGGCTGAACATTTTTC
Nox1 -Reverse	TCGACACACAGGAATCAGGAT
Nox2 -Forward	CCTTTGGTACAGCCAGTGAAGA
Nox2 -Reverse	CAATCCCGGCTCCCACTAACATC
Nox4 -Forward	GAAGGGGTTAAACACCTCTGC
Nox4 -Reverse	ATGCTCTGCTTAAACACAATCCT
eNOS -Forward	GGCTGGGTTTAGGGCTGTG
eNOS -Reverse	CTGAGGGTGTCGTAGGTGATG
Xo -Forward	ATGACGAGGACAACGGTAGAT
Xo -Reverse	TCATACTTGGAGATCATCACGGT
Sod1 -Forward	AACCAGTTGTGTTGTCAGGAC
Sod1 -Reverse	CCACCATGTTTCTTAGAGTGAGG
Cat -Forward	AGCGACCAGATGAAGCAGTG
Cat -Reverse	TCCGCTCTCTGTCAAAGTGTG
Gpx1 -Forward	AGTCCACCGTGTATGCCTTCT
Gpx1 -Reverse	GAGACGCGACATTCTCAATGA
